# Research on the applicability of an exercise rehabilitation app aiming to improve the mental and physical health of breast cancer patients in the post-operative period

**DOI:** 10.3389/fpsyg.2023.1126284

**Published:** 2023-06-30

**Authors:** Jiaxin Zhu, Hu Niu, Dianjie Lu, Yuqi Li, Meng Ding

**Affiliations:** ^1^College of Physical Education, Shandong Normal University, Jinan, China; ^2^Department of Breast and Thyroid Surgery, Jinan Central Hospital affiliated to Shandong First Medical University, Jinan, China; ^3^School of Information Science and Engineering, Shandong Normal University, Jinan, China; ^4^Jinan Zhensheng School, Jinan, China

**Keywords:** breast cancer, radiotherapy or chemotherapy period, exercise rehabilitation, telerehabilitation, applicability

## Abstract

**Purpose:**

Breast cancer is one of the most common malignant cancers in women, seriously endangering the physical and mental health of patients. In this study, we developed an app for breast cancer patients undergoing radiotherapy or chemotherapy with a focus on exercise interventions, supplemented by nutritional and psychological interventions, to verify the applicability of the app for these patients and its impact on their quality of life, sleep, and psychological state. We also investigated the patients’ experience and perceptions of the app.

**Methods:**

A total of 17 participants, aged 42–58 years, were recruited for this study using a mixed-methods design, including quantitative group pre-and post-test scores and qualitative interview results. The participants used the app for 8–18 weeks depending on their radiotherapy or chemotherapy cycle. During the radiotherapy or chemotherapy period, the participants used the “Yun Dong Ru Kang” exercise rehabilitation app to perform aerobic exercises twice a week, as well as rehabilitation exercises appropriate to their radiotherapy or chemotherapy stage, and used the app on their own the rest of the time. The primary results included their scores on the PSSUQ overall assessment usability questionnaire, the users’ use of the app, and the results of the interviews; the secondary indicators were quality of life, sleep status, and anxiety and depression status.

**Results:**

An overall score of 6.2 (out of 7 points) on the PSSUQ questionnaire indicates the high usability; the average use time per subject per week was 97.69 ± 11.82 min, which exceeds the minimum use time, but the average use time tended to decrease as the use time was postponed. Promoted articles on nutritional diets received the most hits. The results of the interviews were consistent with the questionnaire scores, with the majority of participants believing that the means of exercise should be enriched and the interface optimized, while the reduction in the length of use was related to the participants’ own state of learning about calisthenics. In the results of the Breast Cancer-Specific Scale FACT-B, there was a significant increase (*p* < 0.05) in the Emotional Status dimension score and a significant decrease (*p* < 0.05) on the Additional Concerns dimension score. In the results of the Pittsburgh Sleep Quality Inventory PSQI, there was a non-significant improvement in all items except for a significant increase (*p* < 0.05) for the Hypnotic Medication item. In the Hospital Anxiety and Depression Scale (HADS), there was no significant improvement in any of the anxiety and depression factors.

**Conclusions:**

The “Yun Dong Ru Kang “app has certain applicability, and the use of the exercise rehabilitation app may effectively reduce the negative impact of chemotherapy side effects on the quality of life, sleep and depression of breast cancer patients in the chemotherapy or radiotherapy phase. Before it is put into use in the future, the app should be enriched with exercise tools, the interface should be optimized, and articles on nutrition and diet should be promoted.

## 1. Introduction

Breast cancer is the most prevalent malignancy among women worldwide and the most common cause of cancer deaths among women in China (Youlden et al., 2012; Bray et al., 2018). The global cancer statistics for 2020 show that there were more than 2.26 million new breast cancer cases and ~685,000 deaths worldwide, with high incidence and mortality rates in most countries (Fitzmaurice et al., 2015; Ding et al., 2020; Sung et al., 2021). However, with the continuous improvement and refinement of treatment and the spread of mass screening for breast tumors in the early stages, there is a clear trend toward higher survival rates, with some data showing that the median 5-year survival rate for Chinese breast cancer patients has reached 88%. There are a number of serious issues that need to be addressed and controlled for during post-operative treatment, e.g., post-operative complications such as physical dysfunction, lymphoedema of the upper limbs, symptoms such as fatigue and sleep disturbances, low psychological mood, and social difficulties due to changes in self-image after surgery. Evaluation of a patient’s functional status during radiotherapy or chemotherapy plays a crucial role in the choice of subsequent treatment options and in the early return to a social role.

Exercise interventions for breast cancer patients have been shown to improve quality of life and emotional state after breast cancer treatment, and have a palliative effect on common post-treatment adverse symptoms. Exercise intervention rehabilitation can effectively improve the mental health and physical activity levels of radiotherapy or chemotherapy breast cancer patients, facilitate the return to work of post-operative patients, and better improve the quality of life of cancer patients after surgery. Most breast cancer patients experience lymphoedema during the post-operative recovery period (Cheville et al., 2003; Hayes et al., 2012; Shaitelman et al., 2015). Post-operative exercise rehabilitation for breast cancer patients reduces the circumference of the patient’s arm by reducing the thickness of the subcutaneous tissue and enhancing lymphatic drainage, increases the patient’s muscular endurance, improves lymphatic flow to the upper limb and improves upper limb function, which may be associated with a significant improvement in cardiopulmonary endurance. Therefore, post-operative exercise interventions can maintain and restore somatic function and healthy body composition in the affected arm and effectively control the development of lymphoedema (Hasenoehrl et al., 2020). Post-operative exercise interventions for breast cancer patients can also have a positive effect on the alleviation of cancer-related fatigue in radiotherapy or chemotherapy breast cancer patients (Al-Majid et al., 2015), and several studies have shown that yoga practice can effectively inhibit the growth of inflammatory factors in cancer (Bower et al., 2014; Taso et al., 2014; Vardar Yağlı et al., 2015). This may be related to the production of large amounts of skeletal muscle-derived IL-6 during exercise, which inhibits the secretion of IL-1β and TNF-α, the main substances that cause fatigue, while promoting the secretion of beta-endorphins from the pituitary gland and improving the body’s tolerance. In addition, Rogers et al. (2017) found that exercise reduces postoperative sleep disturbance in breast cancer patients through a physical activity intervention. Long-term, sustained, moderately intense aerobic exercise repairs the function of the immune system, which in turn promotes the restoration of organ function and helps to reduce adverse somatic reactions, thereby improving sleep disorders and improving the quality of life. Most patients’ perceptions of breast cancer, prognosis, long-term treatment-related side effects, and fear of cancer recurrence trigger anxiety and depression. It has been demonstrated that post-operative exercise interventions for the rehabilitation of breast cancer patients can improve breast-cancer-related health outcomes, including anxiety and depressive symptoms (Campbell et al., 2019). However, there are relatively few studies related to exercise interventions in the post-operative radiotherapy or chemotherapy period.

With the popularity and widespread use of the Internet, providing exercise interventions, nutritional support, and psychological interventions for patients through smartphone apps has become possible. The results of a large number of global studies have shown that interventions for patients through smartphone apps, online platforms, and online video calls can effectively improve the quality of life and physical function of breast cancer patients during the recovery period following treatment (Gokal et al., 2016; Kourbelis et al., 2018; Hayes et al., 2019). These results verify the effectiveness, applicability, and acceptability of smartphone apps. Several studies have been conducted on different forms of exercise rehabilitation interventions for breast cancer radiotherapy or chemotherapy patients in various ways through different apps, with results showing improved mental health, quality of life, and physical exercise levels of breast cancer patients (Lyons et al., 2016; Coughlin et al., 2017). The effectiveness, usability, convenience, and practicality of the apps have also been verified (Xiaosheng et al., 2018; Sotirova et al., 2021).

Currently, the functions of breast cancer mobile medical apps in the market are mainly to monitor and manage symptoms during chemotherapy and to provide health education on diet and nutrition and psychological aspects. As people’s lives become more intelligent, this gap in the field of mobile health care should be improved as soon as possible to provide patients with the possibility to enjoy mobile health care safely and efficiently. This study has developed an app for breast cancer patients undergoing radiotherapy or chemotherapy, which is based on exercise intervention and supplemented with nutritional and psychological interventions, providing users with professional personalized exercise and health guidance, sound dietary advice, and relief of their negative emotions. Users simply register on the internet and log in directly to the App operating system, then use the App’s resources on diet and nutrition advice and mental health education to improve their post-breast cancer recovery according to their specific needs. The first objective was to test the applicability of the app for the first time and to explore the patients’ experience and perception of using the app. The second objective was to consider the app’s effect on secondary indicators such as quality of life, psychological status and physical mobility in patients undergoing radiotherapy for breast cancer. Finally, the third objective of this study was to analyze the improvements suggested and implemented by the patients.

## 2. Materials and methods

### 2.1. Participants

This study recruited 15–20 participants from the Qian fo Mountain Hospital in Jinan, Jinan Central Hospital of Shandong First Medical University and Shandong Provincial Hospital of Traditional Chinese Medicine in Shandong Province by physician recommendation.

Inclusion criteria: (1) Women with breast cancer (stage I–III); (2) age ≥ 18 years; (3) no recurrent disease; (4) completed surgery with chemotherapy or radiotherapy + chemotherapy.

Exclusion criteria: (1) Speech or communication impairment; (2) lower limb incompetence; (3) medical contraindications to exercise; (4) diagnosed with a major medical or psychiatric illness (except cancer); (5) cognitive impairment and dementia; (6) inability to use a smartphone by themselves or with the assistance of others.

Participants who met the inclusion criteria were asked to sign an informed consent form for this study and to complete the relevant questionnaire. The study was approved by the College of Physical Education of Shandong Normal University and followed the Declaration of Helsinki recommendations for research involving human subjects (Harriss and Atkinson, 2011).

### 2.2. Experimental procedure

A smartphone app for exercise rehabilitation aimed at breast cancer patients undergoing radiotherapy or chemotherapy, “Yun Dong Ru Kang,” was developed and put into use with breast cancer patients undergoing radiotherapy or chemotherapy to test its applicability. This was a mixed-methods study, with both a single-group pre-and post-test quantitative component and a feedback session and interview qualitative component.

#### 2.2.1. Smartphone app development

Based on the literature and interviews with experts to identify specific interventions for breast cancer patients, the development and testing of an Android and PC-based back-end data management system was carried out in collaboration with students from the School of Information Engineering at Shandong Normal University. Functional testing of continuously delivered sub-modules during the development process ensured the smooth development and application of the software. The development environment was as follows.

1. Developed hardware environment: CPU: Intel(R) Core (TM) i5-9300H CPU @ 2.40 GHz 2.40 GHz RAM: 16.0 GB.
2. Running hardware environment: Intel Pentium IV 3,000 MHz or higher Memory: 1,024 M or higher, 1G or higher hard disk.
3. Operating system where the software was developed: Windows 10.
4. Software development environment/development tools: Hbuilder IDEA openJDK1.8.
5. This software runs on the following platforms/operating systems: Android IOS.
6. Programming language: Javascript HTML.
7. Source program volume: 11179.

#### 2.2.2. Smartphone app design

**Figure 1:**
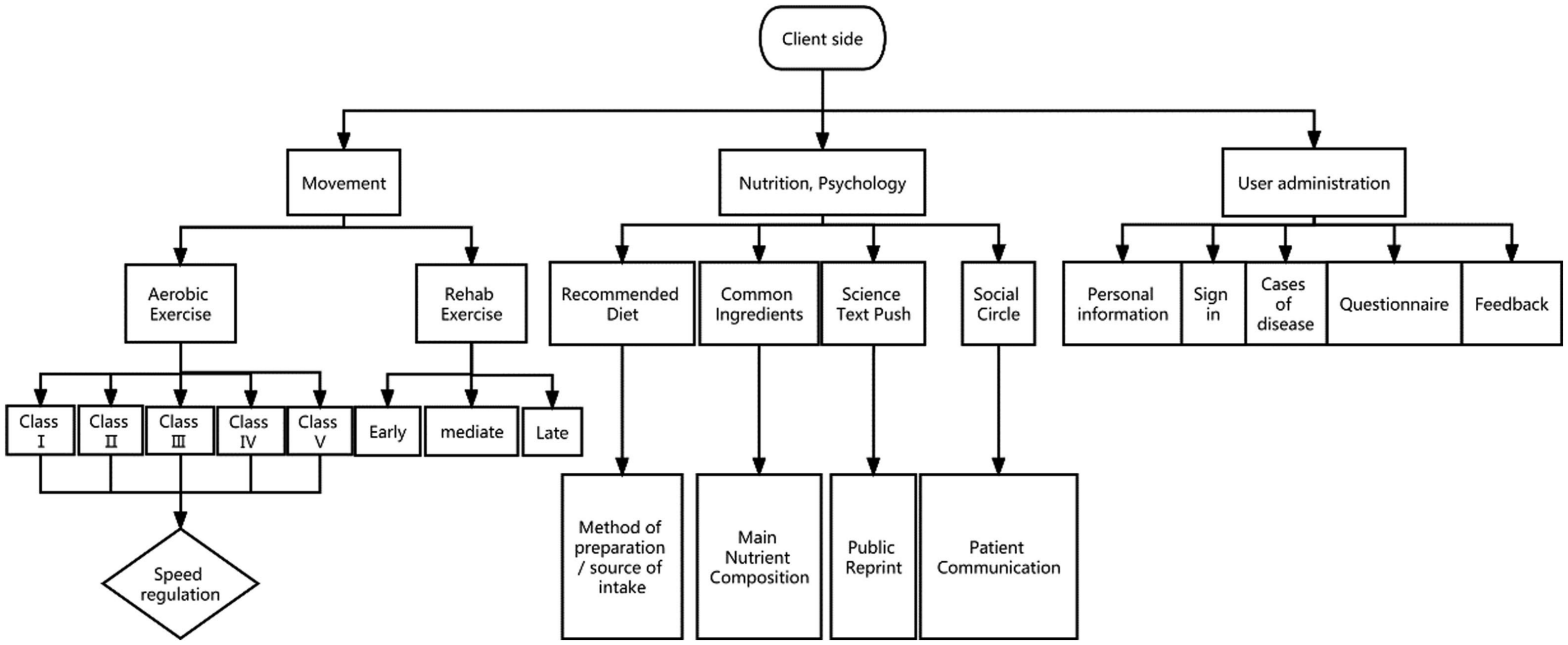
App client operation flow chart.

For ease of operation, the app client was divided into three sub-modules in total: Exercise, nutrition psychology, and user management (Figure 1).

##### 2.2.2.1. Motion module

1. Aerobic exercises

Considering the female subject population and their interest and receptivity to learning motor skills, the exercise intervention for the exercise module was chosen to be moderate-intensity aerobics with an expected exercise intensity of 60–80% of HRmax for 20–40 min according to the American College of Sports Medicine (ACSM) exercise guidelines for cancer survivors (Campbell et al., 2019).

**Figure 2:**
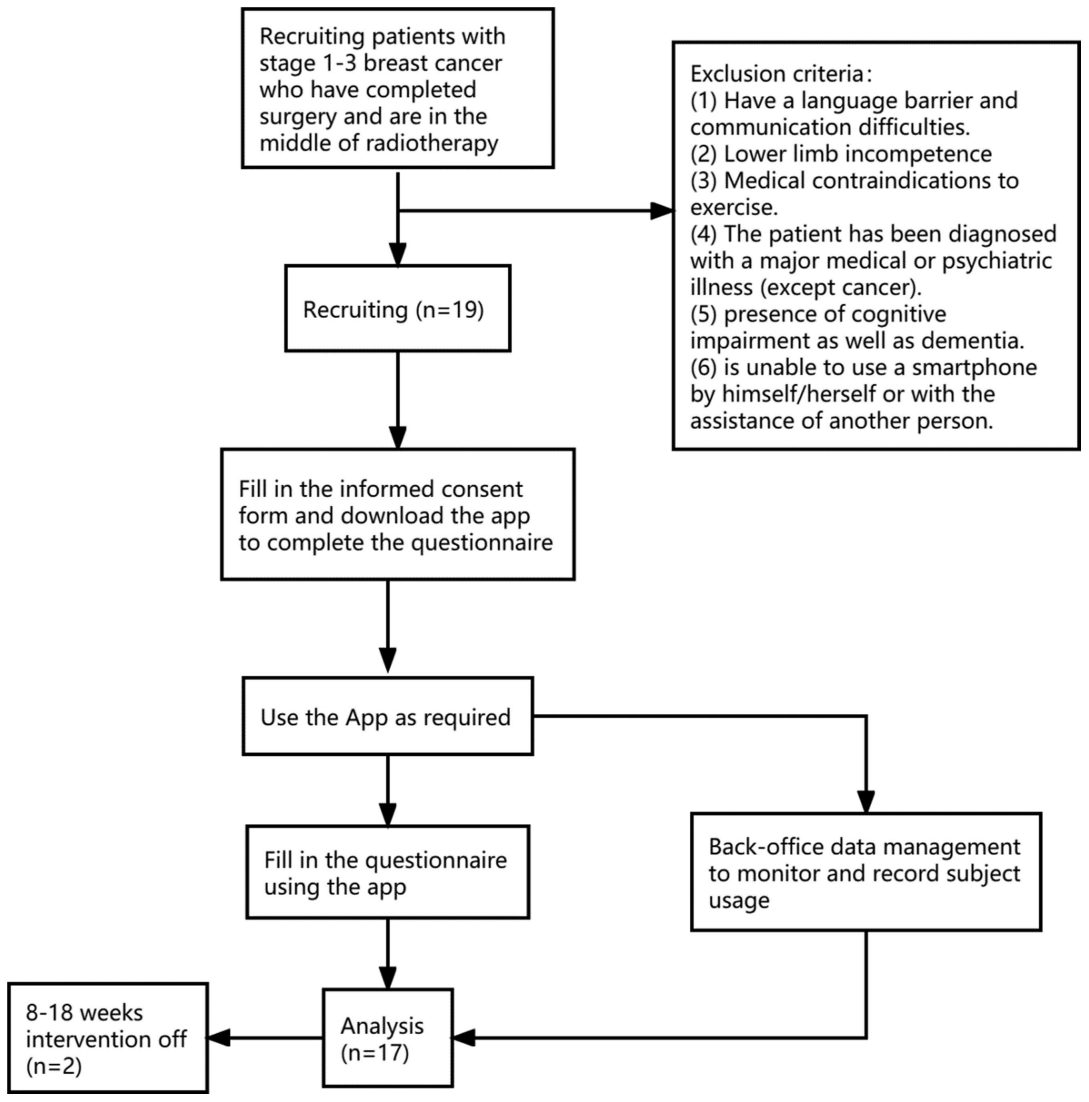
Study flowchart.

Five aerobic exercise instructional videos of 20 min in length were recorded by an aerobic professional based on difficulty (Table 1). Considering the difference in cardiorespiratory fitness between the patients and the instructor, the instructor recorded the videos at 50–60% HRmax (HRmax = 220 − age) exercise intensity and set the low-speed function in the app video playback interface to adjust the video speed according to the patient’s RPE to ensure that the subject exercises at the target exercise intensity in Figure 2.

1. Post-operative breast cancer rehabilitation exercises

**Table 1:** Aerobics difficulty ratings.

Difficulty	Upper limb movements	Lower limb movements	BPM
I	Rarely	No shock + low shock	105–115
II	Less	No shock + low shock	105–115
III	Less	Low shock	105–115
IV	Moderate	Low shock + change of direction	110–120
V	Moderate	Low shock + change of direction	115–125

Post-operative breast cancer rehabilitation exercises are common in hospital clinics and are divided into three sets of rehabilitation exercises for the early, middle, and late stages (Table 2), which are suitable for breast cancer patients from 24 h to 1 month after surgery, 1–3 months after surgery, and 3 months or more after surgery, respectively. Through arm and shoulder exercises, post-breast cancer patients can effectively promote blood and lymphatic fluid return, prevent lymphoedema, promote wound healing, prevent scar adhesions, and improve shoulder mobility.

**Table 2:** Recovery exercises for different stages after breast cancer surgery.

Staging	Applicable groups	Exercise frequency	Exercise time
Early stage	24 h to a month after surgery	Three times daily	10–15 min each
Middle stage	1–3 months after surgery	Three times daily	10–15 min each
Late stage	3 months or more after surgery	Once daily	10–15 min each

##### 2.2.2.2. Nutrition, psychology module

Relevant information was delivered to patients in the form of scientific articles, integrating the retrieved literature related to nutrition, disease, exercise, and treatment, and by preparing soft copy to push to patients. Using social media platforms such as WeChat and Weibo, relevant scientific articles in various fields were reproduced with the author’s permission, so that the patients could have a deeper understanding of their disease and a correct perception of breast cancer, thus overcoming or improving their negative psychological emotions. A social forum for patients was set up, where they could post updates and pictures at any time to facilitate patient-to-patient communication.

### 2.3. Intervention programs

Subjects were screened for pre-exercise health *via* the Physical Activity Adaptation Questionnaire (PAR-Q) prior to the end of chemotherapy or radiotherapy after surgery, and were contacted by the researcher and completed an informed consent form.

Patients need to install the app and understand how to use it with the help of the researcher and to record chemotherapy or radiotherapy cycles: On Day 1 of the first course of radiotherapy or chemotherapy, the patients filled in personal information on the app and completed questionnaires on quality of life, sleep quality, anxiety and depression, and initial physical measurements; the data were recorded and analyzed by the back-end data center; on Days 2–21 (for a 21-day course of radiotherapy or chemotherapy, for example), information (on exercise, nutrition, and psychological support) was pushed to the patient twice a week *via* the app according to the subject’s radiotherapy or chemotherapy cycle, and exercise reminders were set. For the remainder of the time, the patients browsed the content in the app on their own and participated in patient interactions, etc.

The study duration of this study depends on the subject’s chemotherapy or radiotherapy treatment (ranging from 8 to 18 weeks). Questionnaires on quality of life, sleep quality, anxiety and depression, and system suitability are completed *via* the app and recorded by the back office after the subjects have completed all chemotherapy or radiotherapy sessions.

### 2.4. Evaluation indicators

The main evaluation indicators were the satisfaction rating of the participants with the “Cloudy Rooster” sports rehabilitation app and the use of the app by the participants, with both measured after the intervention and recorded for analysis. Satisfaction was recorded using the Post-study System Usability Questionnaire (PSSUQ). The participants’ usage was recorded and analyzed using the PC-based back-end data management system on the participants’ usage time, frequency, and article view counts. Secondary indicators were quality of life, sleep quality, and anxiety and depression status, measured and recorded at baseline (first application of the app) and after the intervention. Quality of life indicators were measured using the Breast Cancer-Specific Scale FACT-B (version 4.0); sleep quality indicators were measured using the Pittsburgh Sleep Quality Index (PSQI); anxiety and depression were measured using the Hospital Anxiety and Depression Scale (HADS).

#### 2.4.1. Satisfaction rating

The overall evaluation usability questionnaire PSSUQ was used to assess the user’s perceived satisfaction with a system or program (Al-Tahat, 2021). The PSSUQ contains 16 rating entries with four rating dimensions: System quality, information quality, interface quality, and overall rating, using a 0–7 rating scale from strongly disagree to strongly agree; the higher the score, the higher the usability.

#### 2.4.2. Utilization indicators

The PC back-end data management system maintained statistics on the number of times the sports instructional videos were played, the number of times the users logged into the app, the cumulative amount of time the app was used, and the number of views of the pushed articles.

#### 2.4.3. Quality of life indicators

The FACT-B (version 4.0) is a specific scale for measuring the quality of life of breast cancer survivors with good reliability, validity, and responsiveness (Imayama et al., 2013; Chang and Guo, 2015). The FACT-B (version 4.0) includes five dimensions: Physical status (seven entries), social/family status (seven entries), emotional status (six entries), functional status (seven entries), and additional concerns (nine entries); it uses a five-point scale of not at all, a little, somewhat, quite, and very. The FACT-B (version 4.0) specific scale is scored on a direct scale of 0–4 for positive entries and a reverse scale for negative entries, with all scores ranging from 0 to 144, with higher scores indicating better quality of life.

#### 2.4.4. Sleep quality indicators

The PSQI is used to assess the sleep quality of patients with sleep disorders, sick leave sleep quality in patients with mental disorders, and sleep quality in the general population (Sancho-Domingo et al., 2021). The scale contains 19 self-assessed items grouped into seven components: Subjective sleep quality, sleep latency, sleep duration, habitual sleep efficiency, sleep disorder accrual problems, sleep medication use, and daytime dysfunction. Points are awarded according to the response to entry 7, with 0 points for “none,” 1 point for “<1 week/time,” 2 points for “1 to 2 weeks/time” and 3 points for “≥3 weeks/time.” The results of the questionnaire are scored with reference to the PSQI scoring instructions, with higher scores indicating worse quality of sleep.

#### 2.4.5. Anxiety and depression indicators

The HADS is used to assist doctors in assessing the levels of anxiety and depression in hospitalized patients (Annunziata et al., 2020). The HADS consists of 14 items to calculate the patient’s composite score, seven of which are anxiety-related and the other seven are depression-related. A four-point scale is used, with each question scoring 0–3, which means 21 points each for anxiety and depression, 0–7 for no symptoms, 8–10 for suspicious presence, and 11–21 for definite presence.

### 2.5. Statistical methods

The results of this pilot study were processed using SPSS 22 for statistical analysis. The Shapiro–Wilk test was chosen to test the normality of the sample, and it turned out that this sample did not obey a normal distribution. Therefore, the measures were compared using two independent samples non-parametric test (Mann–Whitney *U*) to compare the difference before and after the intervention. *p* < 0.05 indicates that the difference is statistically significant. Cohen’s d-value was chosen as the effect size to observe the effect size and was calculated as follows.

Cohen'sd=M2−M1/SDpooled

$\mathrm{SDpooled}=\surd \left(\left(SD12+SD22\right)/2\right)$

Where *M* is the mean and SD is the standard deviation. 0.2 < *d* < 0.5 has a small effect; 0.5 < *d* < 0.8 has a medium difference; *d* > 0.8 has a large effect.

## 3. Results

### 3.1. Participants

A total of 19 participants were recruited through their doctors’ recommendations and Internet publicity. During the course of the trial, two participants dropped out for personal reasons, while the other 17 participants persisted in completing the study. The results from these 17 participants were included in the data analysis.

Table 3 presents the basic information of the participants: Their mean age was 49.82 ± 4.25 years, 76.5% had a high school education or above, all were married, 76.5% were employed, 17.6% had stage I breast cancer, 70.6% had stage II breast cancer, 11.8% had stage III breast cancer, 58.8% were undergoing surgery + chemotherapy, and the rest were undergoing surgery + chemotherapy + radiotherapy.

**Table 3:** Basic information about the participants.

Category	*N* (*n* %)/(X ± SD)
Age (years)	49.82 ± 4.25
Duration of chemotherapy or radiotherapy (weeks)	12.59 ± 2.69
Educational level	
Junior high school	4 (23.5)
High school and post-secondary	7 (41.2)
Tertiary	4 (23.5)
Bachelor and above	2 (11.8)
Marital status	
Married	17 (100)
Occupational status	
In-service	13 (76.5)
Retirement	4 (23.5)
Cancer stages	
Period I	3 (17.6)
Period II	12 (70.6)
Phase III	2 (11.8)
Type of surgery undergone	
Radical Breast Cancer Surgery	9 (52.9)
Excision of masses	6 (35.2)
Others	2 (11.8)
Treatment modalities	
Surgery + chemotherapy	10 (58.8)
Surgery + chemotherapy + radiotherapy	7 (41.2)
Location	
Jinan City	5 (29.4)
Liaocheng City	3 (17.6)
Heze City	2 (11.8)
Linyi City	3 (17.6)
Qingdao City	4 (23.5)

### 3.2. App suitability analysis

At the end of the trial, a total of six of the 17 participants agreed to be interviewed. Some of the subjects were not interviewed for a number of reasons. Objective reasons may be that the patients were unable to attend the interview on the appointed date due to family or work reasons, or that they had treatment arrangements on the same day, or that they were too weak after chemotherapy or radiotherapy to be interviewed; subjective reasons may be that some of the patients were introverted, not good at expressing themselves or did not think that attending the interview would be very helpful to their condition.

#### 3.2.1. Overall usability evaluation analysis

After finishing radiotherapy or chemotherapy, the participants filled out the overall assessment usability questionnaire on the “Yun Dong Ru Kang” app, with the following results: System quality score of 6.6; information quality score of 5.9; interface quality score of 5.9; overall score of 6.2.

#### 3.2.2. Back-office user usage data analysis

The data collected from the back-end data management system during user use showed that the average time that the users spent using the app during the intervention period was 97.69 ± 11.82 min/week use, and the average time spent using the app by this segment was 99.18 ± 8.39 min/week; the number of users with 8, 15, and 18 weeks of use was two each and the average time spent using the app by these segments was 116.44 ± 2.69, 89.93 ± 1.87, and 78.56 ± 1.28 min/week, respectively.

#### 3.2.3. Quality of life indicator analysis

As shown in Table 4, two independent sample tests were conducted on the FACT-B scale completed after the participants finished radiotherapy or chemotherapy and the FACT-B scale scores before the use of the “Yun Dong Ru Kang “app. The results showed that there was no significant difference between the pre-and post-scores of the 17 subjects on physical status, social and socio-familial situation, emotional status, functional status, additional focus and total score (*p* > 0.05, *d* < 0.8).

**Table 4:** Breast cancer specificity scale FACT-B index correlation score Mann–Whitney *U* test (*n* = 17).

FACT-B	Groups	Rank mean	*Z*	Value of *p*	*d*
Physiological status	Pre-test	16.12	−0.833	0.405	0.168
	Post-test	18.88			
Socio-familial situation	Pre-test Post-test	16.85	−0.387	0.699	0.249
		18.15			
Emotional status	Pre-test Post-test	15.68	−1.116	0.264	0.218
		19.32			
Functional status	Pre-test	17.32	−0.104	0.917	0.057
	Post-test	17.68			
Additional focus	Pre-test	19.91	−1.47	0.142	0.556
	Post-test	15.09			
Total score	Pre-test	16.79	−0.415	0.678	0.0982
	Post-test	18.21			

#### 3.2.4. Sleep quality indicator analysis

As shown in Table 5, two independent sample tests were performed on the PSQI scale completed after the participants finished chemotherapy or radiotherapy and the PSQI scale scores before the use of the “Yun Dong Ru Kang “app. The results obtained showed no significant differences in sleep quality, time to asleep, sleep time, sleep efficiency, sleep disorders, daytime dysfunction and pre and post scores of total scores (*p* > 0.05, *d* < 0.8) and a significant increase in hypnotic drug scores (*p* < 0.05; *d* > 0.8).

**Table 5:** Pittsburgh sleep quality index PSQI correlation score Mann–Whitney *U* test (*n* = 17).

PSQI	Groups	Rank mean	*Z*	Value of *p*	*d*
Sleep quality	Pre-test	18.71	−0.787	0.431	0.265
	Post-test	16.29			
Time to sleep	Pre-test	17.65	−0.098	0.922	0.1
	Post-test	17.35			
Sleep time	Pre-test	17.91	−0.273	0.785	0.096
	Post-test	17.09			
Sleep efficiency	Pre-test	18	−0.348	0.728	0.12
	Post-test	17			
Sleep disorders	Pre-test	17	−0.338	0.735	0.117
	Post-test	18			
Hypnotic drugs	Pre-test	15	−2.385	0.017*	0.873*
	Post-test	20			
Daytime dysfunction	Pre-test	19.24	−1.101	0.271	0.256
	Post-test	15.76			
Total score	Pre-test	18.18	−0.413	0.68	0.194
	Post-test	16.82			

*p < 0.05 or *d* > 0.8.

#### 3.2.5. Anxiety and depression indicator analysis

As shown in Table 6, two independent sample tests were conducted on the HADS scale completed after the participants finished chemotherapy or radiotherapy and the HADS scale scores before the use of the “Yun Duo Ru Kang” app, and the results showed that there was no significant difference between the pre-and post-anxiety and depression scores (*p* > 0.05, *d* < 0.8).

**Table 6:** Hospital anxiety depression scale HADS correlation score Mann–Whitney *U* test (*n* = 17).

HADS	Groups	Rank mean	*Z*	Value of *p*	*d*
Anxiety	Pre-test	17.79	−0.175	0.861	0.044
	Post-test	17.21			
Depression	Pre-test	17.74	−0.14	0.889	0.04
	Post-test	17.26			

## 4. Discussion

The PSSUQ scale used to evaluate the usability of the app has four dimensions with scores of 6.6, 5.9, 5.9, and 6.2, respectively. The quality of the system was highly appreciated by most participants, while a few of them thought that the functions of the “Yun Dong Ru Kang” app were relatively simple and uninteresting. The low rating for information quality is mainly reflected in item 7: “The system gives me clear instructions on how to solve the problem,” which has a mean score of 2.18, which is somewhat different from the mean score of 5.9 for information quality, which is also found in other scholars using this questionnaire scored too low. After discussions with the app developers, the low rating for the quality of information is due to the fact that the app was not developed with “error alerts,” but this did not have a negative impact on the suitability of the app in relation to the ease of use and the results of the interviews. The interface quality dimension also showed low scores, mainly related to the “single color” and “blurred interface” according to the feedback of the participants. With regard to the overall evaluation of the app, the majority of the participants reported that the “aerobics” module in the exercise module had poor picture quality, noisy background music, and too little content. Considering the shortage of research funding at the beginning of the study to provide professional video equipment, studios, etc., this situation could be improved with more funding in subsequent studies.

The subjects spent an average of 97.69 ± 11.82 min per person per week in the app, which exceeded the minimum use of aerobics twice per week, but the average use time tended to decrease as the use time was postponed. One of the reasons for this is that the intervention period was too long. The “Yun Dong Ru Kang” app is relatively simple and the frequency of use decreased as the intervention period grew; another reason is that the exercise module is small and easy to learn, with aerobic and rehabilitation exercises that patients can perform alone without watching the video after several exercises. According to the back-end data management system, the number of users of the “forum” function was low, and participants reported that this was related to the low number of users and their reluctance to communicate with others. All interviewees indicated that they were interested in the articles, with the diet category receiving a significantly higher click-through rate than the other categories. It has been shown that smartphone applications (diet, exercise) through online and mobile lifestyle interventions are safe and feasible for breast cancer patients and that they are effective in reducing weight and waist circumference in a short period of time, even more so if the intervention is personalized to each patient. Therefore, individualized interventions may be the future direction of this study (McCarroll et al., 2015).

The FACT-B scale scores were calculated after the use of the app throughout the chemotherapy or radiotherapy cycle and no significant differences were found between the pre and post scores for physical status, social and family status, emotional status, functional status, additional focus and total score. Of these, the additional focus dimension score d value of 0.556 (0.5 < *d* < 0.8 moderate difference) was a more pronounced decline, which may have been influenced mainly by the patient’s gender self-perception, sexuality, and upper limb oedema, a decline in this dimension score that seems inevitable with radical breast cancer surgery, and chemotherapy or radiotherapy side effects such as hair loss and endocrine disorders. With the exception of the additional focus dimension, the rank mean of all dimensions of the FACT-B scale has increased. Since the inception of chemotherapy or radiotherapy, the impact of its side effects on quality of life has been negative (Park et al., 2015; Travier et al., 2015; Cha et al., 2017). The fact that there was no decrease and a slight improvement in the quality of life of the subjects after chemotherapy or radiotherapy proved that the use of the app during chemotherapy or radiotherapy for breast cancer was effective in alleviating the negative impact of the side effects of chemotherapy or radiotherapy on the quality of life of the patients. It was found that the data on all dimensions of the FACT-B scale did not increase significantly, which may be related to the form of exercise and could be verified in future studies by adding other means of exercise such as resistance exercises as appropriate.

Subjects who used the app throughout the chemotherapy or radiotherapy cycle showed no significant changes in sleep quality, time to sleep, sleep time, sleep efficiency, sleep disorders, daytime dysfunction and overall scores, except for a significant increase in hypnotic drugs scores (pre-test rank mean of “15.00” and post-test rank mean of “20.00”). Some studies have shown that (Imanian et al., 2019; Fox et al., 2020; Kwak et al., 2020) breast cancer patients undergoing chemotherapy or radiotherapy have significant sleep disorders at different stages of treatment compared to before. The mean sleep disorder dimension rank in this study increased, but not significantly, presumably related to hypnotic drug intake. Overall, all factors except for the hypnotic drug dimension and the sleep disorder dimension showed varying degrees of improvement, thus demonstrating that patients can control the growth of sleep disorders by using the “Yun Dong Ru Kang “app during chemotherapy or radiotherapy. Further enrichment of the app to address sleep disorders in the future may lead to better results.

Throughout the whole radiotherapy or chemotherapy cycle, the patients’ anxiety and depression scores on the HADS were not significantly changed by the use of the “Yun Dong Ru Kang” app. By using the app, the patients may have been able to overcome the negative effects of anxiety, such as stress, fear, worry, and panic, caused by the disease and radiotherapy or chemotherapy. Some studies have shown that depression levels in breast cancer patients increase significantly with the duration of radiotherapy or chemotherapy, and, according to the data herein, the patients were able to suppress depression by using the app (Ho et al., 2015; Tsaras et al., 2018; He et al., 2019). However, in terms of the depression factor, the second test showed a higher dispersion than the first test, with some patients experiencing an increase or decrease in depression, but no significant increase overall, suggesting that the use of the app during the radiotherapy or chemotherapy period was able to suppress depression. The results of a previous study on the use of a smartphone app for breast cancer patients to monitor and manage their symptoms during chemotherapy showed that the app reduced the incidence of side effects during chemotherapy and that users reported that the app was effective in recognizing their symptoms (Kearney et al., 2009; McCarroll et al., 2015). The lack of improvement in the patients’ anxiety and depression scores may be related to their lack of information about their illness, which in turn may lead to anxiety. Judging from the status of the patients’ viewing of scientific articles, subsequent enhancement of the frequency and length of disease-and treatment-related articles pushed to patients may be the way to solve this problem.

## 5. Conclusion

The “Yun Dong Ru Kang “exercise rehabilitation app is suitable for post-operative radiotherapy or chemotherapy patients with breast cancer, as it may reduce the negative impact of radiotherapy or chemotherapy side effects on the quality of life, sleep and depression of breast cancer patients undergoing radiotherapy or chemotherapy. Our app, like other mobile health tools, needs to be constantly revised and updated. Therefore, before the “Yun Dong Ru Kang “exercise rehabilitation app is put on the market, it is necessary to optimize the content of the exercise module, improve the quality of the content, and enrich the user’s means of exercise during the radiotherapy phase; optimize the interface UI to improve the user’s experience. During the follow-up management process, the promotion of articles on diet will be increased according to the interest of patients. However, this study also had some limitations. Firstly, the study was not a randomized controlled trial, so the findings need further validation and future studies should include both groups of women and a baseline assessment at the time of participation; secondly, the sample size in this study was small and the App should be tested in a larger sample size in future studies.

In the future, the “Yun Dong Ru Kang” app will be able to add a personal health record column, an experience sharing column and an expert consultation column to enable users to record personal rehabilitation medical-related letters and body-related self-measurement (e.g., arm circumference, joint mobility, sleep time, etc.), view data charts and receive alerts on abnormal data; encourage The user community is encouraged to share their experiences of self-management and living with cancer; experts in breast cancer are invited to regularly contribute to the app to educate the user community and answer their questions. The ‘Yun Dong Ru Kang’ app can be introduced to post-breast cancer patients in different countries by incorporating multiple languages into the interface (e.g., English).

## Data availability statement

The original contributions presented in the study are included in the article/Supplementary material, further inquiries can be directed to the corresponding authors.

## Ethics statement

The studies involving human participants were reviewed and approved by the College of Physical Education of Shandong Normal University. The patients/participants provided their written informed consent to participate in this study. Written informed consent was obtained from the individual(s) for the publication of any potentially identifiable images or data included in this article.

## Author contributions

JZ and MD planned the structure of the manuscript. JZ wrote the first manuscript. MD reviewed and edited the final manuscript. HN revised and adjusted the overall content of the article and helped to draft the manuscript. DL and YL analyzed the data and designed the “Yun Dong Ru Kang” app. All authors contributed to the article and approved the submitted version.

## Funding

This study was supported by the Exercises Promote Health Theory and Practice Innovation Team of Shandong Normal University in China (no. 112/14001).

## Conflict of interest

The authors declare that the research was conducted in the absence of any commercial or financial relationships that could be construed as a potential conflict of interest.

## Publisher’s note

All claims expressed in this article are solely those of the authors and do not necessarily represent those of their affiliated organizations, or those of the publisher, the editors and the reviewers. Any product that may be evaluated in this article, or claim that may be made by its manufacturer, is not guaranteed or endorsed by the publisher.

## Abbreviations

App: Application
PSSUQ: Post-study System Usability Questionnaire
FACT-B: The Functional Assessment of Cancer Therapy – Breast
HADS: Hospital Anxiety and Depression Scale
PC: Personal Computer
HRmax: Exercise Heart rate
RPE: Rating of Perceived Exertion
ACSM: American College of Sports Medicin
PAR-Q: Post-study System Usability Questionnaire
