# Supplementary material for: Research on the applicability of an exercise rehabilitation app aiming to improve the mental and physical health of breast cancer patients in the post-operative period
Source: Front Psychol. 2023 Jun 30;14:1126284. doi: 10.3389/fpsyg.2023.1126284 (PMC10349282; doi:10.3389/fpsyg.2023.1126284)
Supplement: Supplementary file 1 [file Data_Sheet_1.docx]

Supplementary Material

**HOSPITAL ANXIETY AND DEPRESSION SCALE (HADS)**

***Translation to the Chinese language***

**
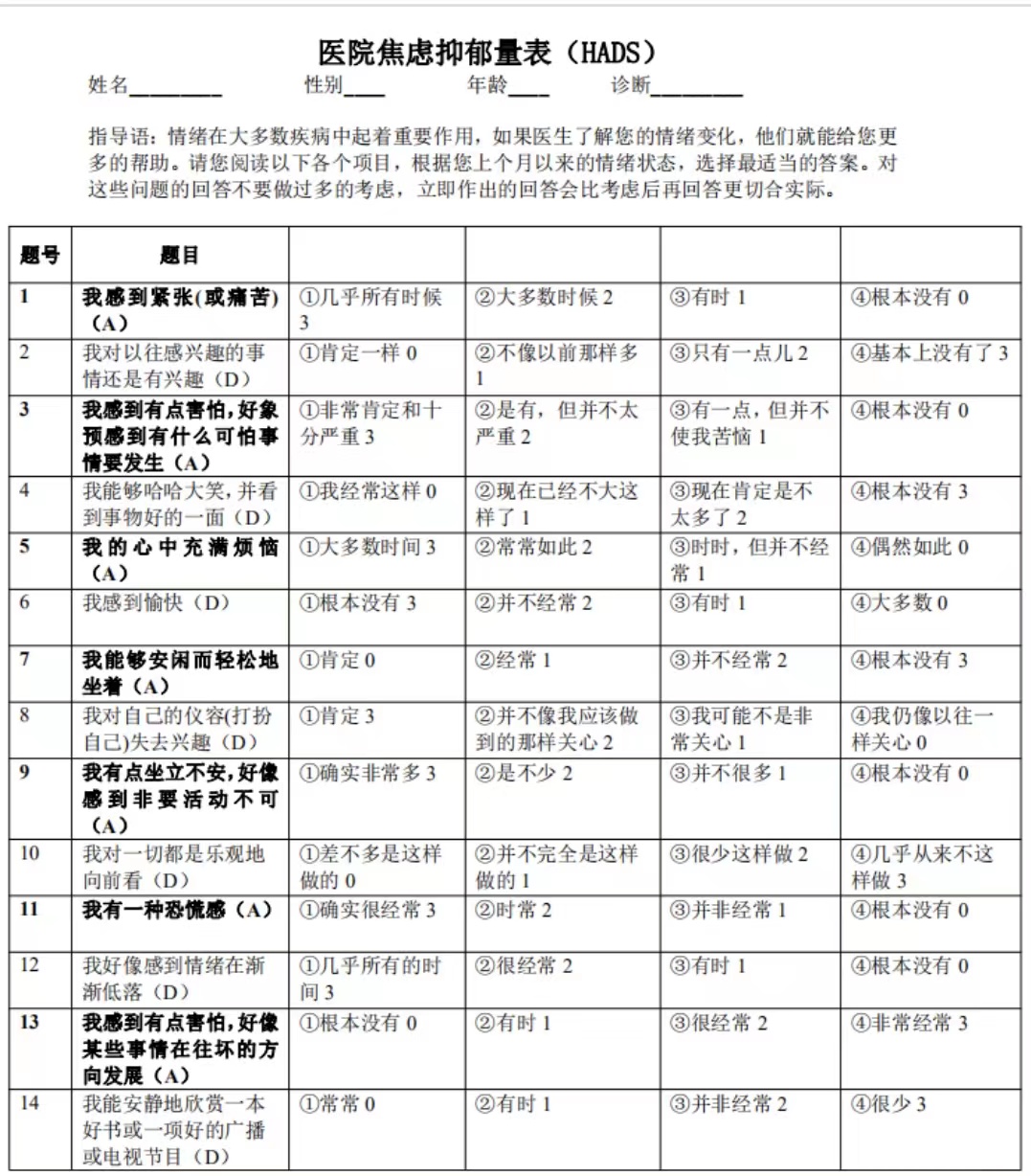
**

**Hospital Anxiety and Depression Scale (HADS)**

***Original version on the English language***

| A | 1. I feel tense or 'wound up': | 8. I feel as if I am slowed down: | D |
| --- | --- | --- | --- |
| 3  2  1  0 | a. Most of the time  b. A lot of the time  c. From time to time, occasionally  d. Not at all | a. Nearly all the time  b. Very often  c. Sometimes  d. Not at all | 3  2  1  0 |
| D | 2. I still enjoy the things I used to  enjoy: | 9. I get a sort of frightened feeling like  'butterflies' in the stomach: | A |
| 0  1  2  3 | a. Definitely as much  b. Not quite so much  c. Only a little  d. Hardly at all | a. Not at all  b. Occasionally  c. Quite Often  d. Very Often | 0  1  2  3 |
| A | 3. I get a sort of frightened feeling as if  something awful is about to  happen: | 10. I have lost interest in my appearance: | D |
| 3  2  1  0 | a. Very definitely and quite badly  b. Yes, but not too badly  c. A little, but it doesn't worry me  d. Not at all | a. Definitely  b. I don't take as much care as I should  c. I may not take quite as much care  d. I take just as much care as ever | 3  2  1  0 |
| D | 4. I can laugh and see the funny side  of things: | 11. I feel restless as I have to be on the  move: | A |
| 0  1  2  3 | a. As much as I always could  b. Not quite so much now  c. Definitely not so much now  d. Not at all | a. Very much indeed  b. Quite a lot  c. Not very much  d. Not at all | 3  2  1  0 |
| A | 5. Worrying thoughts go through my  mind: | 12. I look forward with enjoyment to  things: | D |
| 3  2  1  0 | a. A great deal of the time  b. A lot of the time  c. From time to time, but not too often  d. Only occasionally | a. As much as I ever did  b. Rather less than I used to  c. Definitely less than I used to  d. Hardly at all | 0  1  2  3 |
| D | 6. I feel cheerful: | 13. I get sudden feelings of panic: | A |
| 0  1  2  3 | a. Most of the time  b. Sometimes  c. Not often  d. Not at all | a. Very often indeed  b. Quite often  c. Not very often  d. Not at all | 3  2  1  0 |
| A | 7. I can sit at ease and feel relaxed: | 14. I can enjoy a good book or radio or TV  program: | D |
| 0  1  2  3 | a. Definitely  b. Usually  c. Not Often  d. Not at all | a. Often  b. Sometimes  c. Not often  d. Very seldom | 0  1  2  3 |

**Hospital Anxiety and Depression Scale (HADS)**

*Scoring and interpretation*

**Scoring** *(indicated in the table*):

Anxiety items: 1, 3, 5,7, 9, 11, 13

Depression items: 2, 4, 6, 8, 10, 12, 14

**Total score:**  Anxiety:________ Depression:__________

**Interpretation** *(according to Pais-Ribeiro et al. Neuropsychiatric Disease and Treatment 2018;14;3193-3197)*

| 0-7 | Normal |
| --- | --- |
| od 8 do 10 | Mild |
| od 11 do 14 | Moderate |
| od 15 do 21 | Severe |

**Informed Consent Form**

**
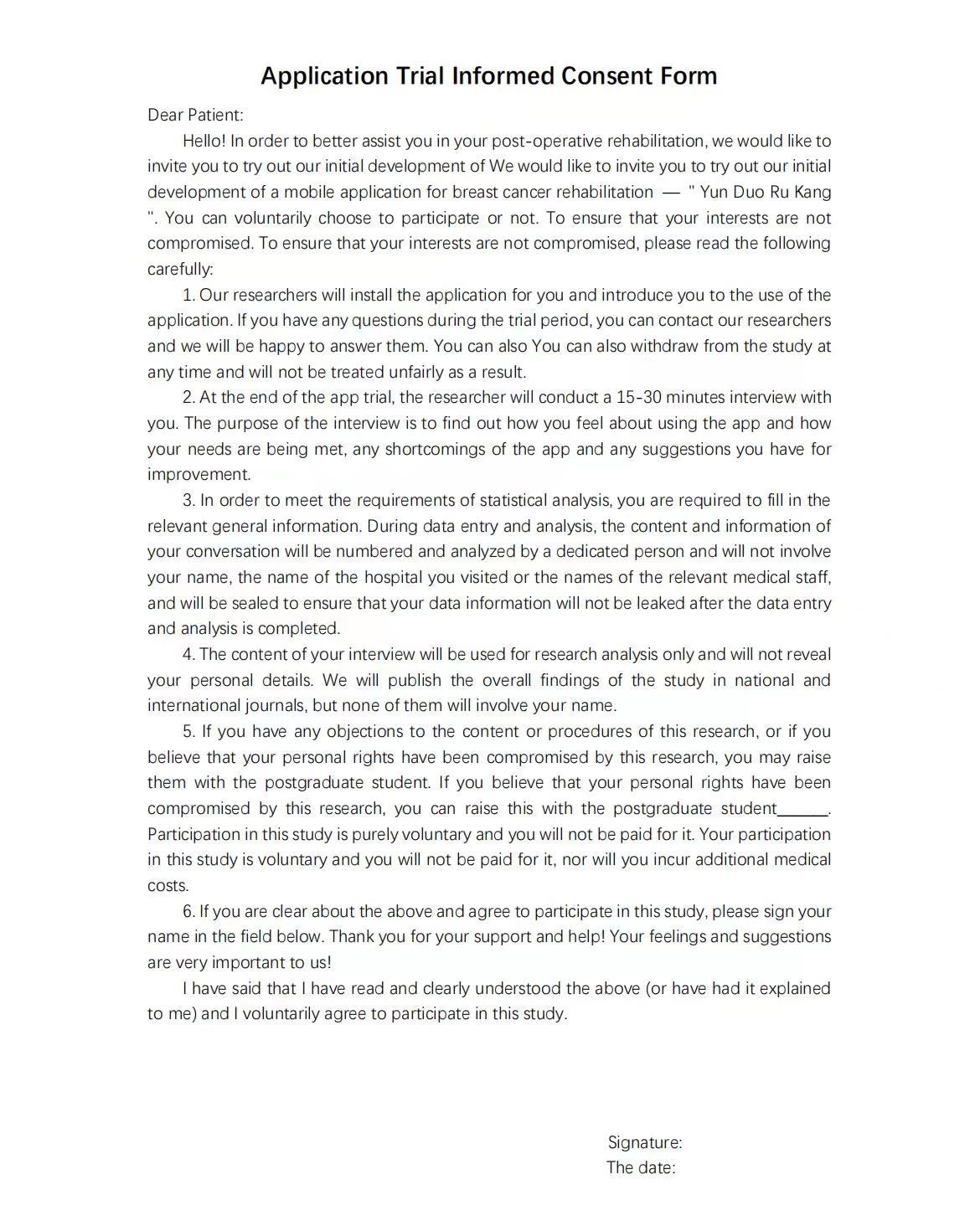
**
